# Pain intensity and pressure pain thresholds after a light dynamic physical load in patients with chronic neck-shoulder pain

**DOI:** 10.1186/s12891-020-03298-y

**Published:** 2020-04-23

**Authors:** A. Grimby-Ekman, C. Ahlstrand, B. Gerdle, B. Larsson, H. Sandén

**Affiliations:** 1 School of Public Health and Community Medicine, Institute of Medicine, Sahlgrenska Academy, University of Gothenburg, Postal address: Box 414, 405 30 Gothenburg, Sweden; 2 Occupational and Environmental Medicine at School of Public Health and community medicine, Institute of Medicine, Sahlgrenska Academy, University of Gothenburg, Gothenburg, Sweden; 3 Pain and Rehabilitation Centre, and Department of Health, Medicine and Caring Sciences, Linköping University, Linköping, Sweden

**Keywords:** Chronic pain, Neck, Shoulder, Pressure pain threshold, Exercise induced hypoalgesia, Work ability

## Abstract

**Background:**

To investigate the development of pain intensity and pressure pain thresholds during and 24 h after a light dynamic physical load among patients with chronic neck-shoulder pain.

**Methods:**

Twenty-six patients with chronic neck-shoulder pain and 12 healthy controls were included. The participants arm-cycled on an ergometer. Effort was rated with the Borg Rating of Perceived Exertion scale (RPE), and pain intensity with an numeric rating scale (NRS). Pressure pain thresholds were measured by an algometer. Participants started a pain diary 1 week before the physical exercise and continued until 1 week after. Pain intensity was assessed before, during and the following two evenings after arm-cycling. Pressure pain thresholds were assessed before, 15 min after, 105 min after and 24 h after.

**Results:**

The chronic pain group showed increased pain intensity during, and the following two evenings after the arm cycling, and decreased pain thresholds immediately after the arm cycling involving painful regions. In the patient group there were no impact on pain thresholds in the neck the following day.

**Conclusions:**

Patients with chronic neck-shoulder pain reported increased pain intensity during and in the evenings after a light dynamic load involving painful regions. In addition, they showed decreased pain thresholds close to the exercise, indicating mechanical hyperalgesia.

## Background

Chronic musculoskeletal pain is a common clinical condition that causes patients to seek medical care and is a major cause of disability and reduced work ability. Clinical experience suggests that some patients with chronic musculoskeletal pain may feel increased pain intensity the day after even light physical exertion. This is important to take into account in work ability assessments. There are some studies showing increased pain intensity and/or increased pain sensitivity (i.e., lowered pain thresholds) in patients with fibromyalgia syndrome and in patients with chronic low back pain during and immediately after exercise [1–4]. In healthy women and men pain thresholds increase during and following exercise, suggesting decreased pain sensitivity [5–7]. Additionally, a study considering patients with whiplash associated disorders (WAD) showed lowered pain thresholds, both locally and remotely, following an exercise program directed at non-painful muscles performing isometric exercises but not in the aerobic cycling exercise [8]. Moreover a study in chronic pain patients revealed that the response of exercise depended on the degree of the pain sensitivity [9]. Thus, there are several studies of patients with chronic musculoskeletal pain showing increased pain thresholds and decreased pain sensitivity during and immediately after exercise which probably can be achieved through activation of endogenous pain inhibitory mechanisms [6, 9, 10]. This latter phenomenon has been termed exercise-induced hypoalgesia (EIH) [7, 11].

A recent review/focus article concluded that studies of the acute effects of exercise on pain sensitivity in patients with chronic pain have shown variable results [11]. Moreover, it was pointed out that most studies have used pressure pain thresholds as a proxy for pain sensitivity and there is a lack of studies using self-reported pain intensity, which is the most important indicator of a pain problem [11]. Furthermore, studies of pain patients and our clinical experience indicate that some patients with chronic muskuloskeletal pain complain of pain the day after light physical exercise, which in turn may suggest a dysfunction of exercise induced pain inhibitory mechanisms or perhaps instead facilitation of pain excitatory mechanisms. To the best of our knowledge, there are no studies that highlight pain intensity and pain sensitivity the day after a light physical exertion in this patient population. Further there are limited knowledge about the temporal pattern of pain intensity and pain thresholds after upper extremity exertion in patients with chronic neck-shoulder pain.

Hence, the main aim of this study was to investigate the development of pain intensity and pain thresholds during and up to 24 h after a light dynamic physical load among persons with chronic neck-shoulder pain and comparing to a control group.

## Methods

Additional descriptions of the design and methods of the study can be found in two previous articles, investigating biomarkers on sub-sets of the subjects in the present study [12, 13]. The overall time schedule for assessments is presented in Table 1.

**Table 1:** Overall time schedule for assessments

	Self-report at home	Assessments at the clinic
**Day 0**		• Standardized medical examination • Baseline questionnaire
**Day 1–6**	Pain diary	
**Day 7**	Pain diary	• **9 am** PPT^1^, NRS^2^, RPE^3^ • **10 am** Arm cycling; NRE, RPE **15 min** after arm cycling**:** PPT, NRS **105 min** after arm cycling: PPT, NRS
**Day 8**	Pain diary	• **9 am** PPT
**Day 9–13**	Pain diary	

^1^Pressure pain threshold, ^2^Numeric rating scale, ^3^ Perceived exertion scale

### Participants

Included were 26 persons (20 women and 6 men) with chronic neck and/or shoulder pain and 12 controls (7 women and 5 men) without ongoing pain. The pain group were recruited from physiotherapy clinics in Region Västra Götaland and from the Occupational and Environmental Medicine clinic, Sahlgrenska University Hospital, Gothenburg, Sweden. The control group were recruited by official message boards at University of Gothenburg, Sweden. Some of the controls were friends or family to the pain group. Included were only subjects between 18 and 65 years of age. The subjects in the pain group could be studying, working or on sick-leave. The controls were all in work or studying.

In order to participate in the study, the patients needed to have musculoskeletal pain lasting longer than 3 month with neck and/or shoulder as main pain localization. The control group had at most 3 days of any pain over the last 12 month.

### Study procedure

The subjects were asked whether they had experienced any symptoms and were examined by an occupational physician at the Occupational and Environmental clinic, Sahlgrenska University Hospital. Most of the the subjects were examined by H. S and a few were examined by a specially trained colleague. The medical examination included a detailed examination of the neck, shoulders, upper extremities, and back in order to check for and detect other diseases. The physician selected who was to be included or excluded in the study. All the included patients had non-specific chronic neck-shoulder pain i.e. no specific pathology could be established.

The participants arm-cycled on an ergometer 1 week after the medical examination. Pain intensity and pressure pain thresholds were assessed before, 15 min after, 105 min after and 24 h after the arm cycling.

The subjects started filling in a pain diary 1 week before the arm-cycling and continued 1 week after.

### Medical examination

The medical examination of the neck and upper limbs was meticulous and followed a specific protocol [12]. In summary, the occupational physician performed a physical examination of the neck and upper limbs (shoulders, elbows, wrists, hands, fingers) which included the succeeding steps: [1] inspection, [2] testing for passive and active motion and range, [3] testing for muscle strength, muscle contraction and pain, [4] palpation of joints, muscle tendons, and insertions, [5] bedside neurologic examination, containing sensory exam in hands/fingers assessing different kinds of sensation, including pinprick, light touch (soft brush), and temperature (rolltemp, one roller being cold and the other warm), muscle stretch reflexes (biceps, triceps, brachioradialis, achilles), and [6] specific tests: cervical spine Lasègue, Spurling’s test (neck compression test), Roos test (abduction external rotation test), bursa test for shoulder bursitis, pronator compression test, Finkelstein’s test, and palpation at the arcade of Frohse.

The subjects who had been diagnosed with fibromyalgia, traumatically-induced neck pain (whiplash), rheumatic or metabolic disease, neurological disease, had symptoms of joint involvement or tendinitis in the shoulder joint or had a severe mental disorder were excluded from the study.

To sum up, the occupational physician selected who was to be included after having taken the medical history and after having completed the clinical examination. The included patients all had non-specific chronic neck pain i.e. no specific pathology was recognized.

### Base survey

On the day of medical examination, the subjects answered a questionnaire including pain drawing, pain intensity [14], kinesiophobia, sleep quality, medication, sick leave, social support, and lifestyle. Furthermore the questionnaire also contained the Shirom–Melamed Burnout Questionnaire (SMBQ) [15–17]. Higher values point towards higher degree of burnout symptoms. Additionally the Hospital Anxiety and Depression Scale (HADS) [18] was included in the questionnaire. A score of 8–10 indicates a possible case, and 11–21 indicates a definite case [18].

### Diary

One week before the physical excerice, the subjects started filling in a pain diary about the evening pain intensity (Numeric Rating Scale; NRS), self reported consequences of pain, pain drawing i.e. spatial distribution of pain, sleep (Karolinska Sleep Diary) [19], activity, mood [20] and medication. The subjects continued to fill in the diary for 1 week after the load.

### Dynamic load: arm cycling

The physical exercise (arm cycling) started at 9 a.m. for all subjects and was performed on an arm-cycle ergometer (Monark Cardio Rehab 891E, Vansbro, Sweden) in order to expose the painful regions to a light dynamic physical provocation. Arm-cycling has formerly been tested by members of the research group in patients with work-related neck pain (FAS Dnr 2008–0755). The subjects in this study arm-cycled for 30 min with a constant pace of 25 laps/min. The arm-cycling began with a load of 100 g for women and 200 g for men. After 10 min, the load was increased to 300 g for women and to 400 g for men. After further 10 min, the load was increased to 500 g for women and to 600 g for men. The participants then arm-cycled with this load for further 10 min. The heart rate was recorded by a chest belt (Actiheart, Camntech, Cambridge, UK).

The reason we chose this load is because in a workplace, in for example an industrial unit, it is quite common for all workers to work with the same tools and under the same load, regardless of individual physique. However, sometimes there is a difference in the load between women and men. In summary, as far as possible, we wanted to emulate real work load and/or work ability assessments.

### RPE and NRS: estimation of effort and pain

Before, after and regularly during the dynamic load, the subjects was told to rate their effort with the Borg RPE scale, and their pain with an NRS scale (numeric rating scale). Borg RPE scale is a 15-point scale, ranging from 6 to 20 (corresponds to an approximate heart rate between 60 and 200), designed to estimate how heavy and strenuous the work is perceived [21]. Effort is defined as a feeling, mainly of fatigue in the muscles, and shortness of breath. Pain intensity was measured using a 11-point NRS scale.

### Algometry: pressure pain thresholds

Pressure pain threshold (PPT) was measured by a hand held electronic pressure algometer (Somedic AB, Hörby) and measured in a standardized manner according to experimental protocols used in previous studies [22, 23]. The contact area was 10 mm and the pressure was applied at a rate of 30 kPa/second. The participants were instructed to mark the PPT by pressing a signal button when they felt the first sensation of pain. Pressure pain thresholds were determined bilaterally at three points along the upper part of Trapezius. The three points at the Trapezius muscle were marked on a line stretching from C7 to the acromion; T1, T2, T3 with T1 being the most medial point. At a maximum value of 800 kPa the measurement was interrupted to avoid bruising and soreness induced by the measurement method.

### Statistical analyses

All analyses were performed using the data program SAS (Statistical Analysis Software, Ver 9.4). Descriptive statistics for the two groups of subjects are presented in Table 2.

**Table 2:** Demographics, comorbidity and pain characteristics at baseline among the control persons and the persons with chronic neck pain. SD = standard deviation, IQR = Interquartile range

***N*** = 38	Chronic neck pain				Control			
	n	Mean	Median	Min, Max	n	Mean	Median	Min, Max
		(SD)	(IQR)			(SD)	(IQR)	
**Age (mean)**	26	51	52.5	23,66	12	34.6	27	22, 61
		[5, 12]	(47, 63)			(16,0)	(23, 53.5)	
**Sex (%)**								
Women	19	73			7	60		
Men	7	27			5	40		
**Education (%)**								
Elementary school	4	15			0	0		
High school	9	35			6	50		
College, university	13	50			6	50		
**Physical activity (%)**								
1 Sedentary	1	4			1	8		
2 Moderate	15	58			4	33		
3 Regular	9	35			5	42		
4 Intense	1	4			2	17		
**Smoking (%)**	4	16			0	0		
**Alcohol consumption (> 9 units/week)(%)**	5	19			4	33		
**HADS – Anxiety**	25	1.2	1.1	0.71, 2.00	12	1.2	1.1	0.71, 1.86
		(0.40)	(0.86, 1.57)			(0.32)	(0.93, 1.29)	
**Work percentage**	26	54	77.5	0, 100	12	88	100	5, 100
		(47.2)	(0, 100)			(29.8**)**	(100, 100)	
**Sick leave or sickness benefit**	26	26	0	0, 100	12	0	0	0, 0
		(43.9)	(0.0,75.0)			(0.0)	(0, 0)	
**SMBQ**	25	1.6	1.6	1.14, 2.55	12	1.4	1.5	0.64, 1.86
		(0.37)	(1.32, 1.86)			(0.33)	(1.32, 1.55)	
**Sleep Quality (insomnia)**	26	3.8	4.5	1.25, 5.25	12	4.6	4.6	3.75, 5.25
		(1.30)	(2.50, 4.75)			(0.56)	(4.25, 5.00)	
Awakening index	26	4.2	4.5	1.33, 6.00	12	4.7	5.0	1.67, 5.67
		(1.32)	(3.00, 5.33)			(1.04)	(4.67, 5.33)	
Sleepiness/fatigue	26	4.3	4.5	2.20, 6.00	12	4.6	4.6	3.00,6.00
		(1.02)	(3.60, 5.00)			(0.91)	(4.20, 5.00)	
**Pain sites**	26	10	9	0,23	4	1.75	1.5	1,3
		(6.26)	(6.0, 13.0)			(0.96)	(1.0,2.5)	

To investigate the development of pain intensity and pressure pain thresholds and the association with the provocation three regression analyses were used:

1. Pain intensity during the provocation. Pain intensity over time, 30 min with assessment every second minutes, is modeled. This is the short-term/acute effect of the provocation on pain intensity.
  Statistical model of pain during the provocation, with load as a factor and time as a covariate
  $$ {Y}_{ij}=\mu +{\alpha}_i+{b}_1\ast {minute}_j+{b}_2\ast {group}_i+{b}_3\ast {minute}_j\ast {group}_i+{\varepsilon}_{ij} $$

The indexes: i = person, j = minute (0, 2, 4, 6, 8, 10, 12, …, 26, 28, 30).

2. Daily pain intensity over time, using the week before (will also be able to estimate within and between individual variation in the sample), the provocation day and the day after.

This is the long-term/24-h effect of the provocation. Day as a factor.

Statistical model of evening pain, with day as a factor

$$ {Y}_{ij}=\mu +{\alpha}_i+{b}_1\ast {day}_{ij}+{b}_2\ast {group}_i+{b}_3\ast {day}_{ij}\ast {group}_i+{\varepsilon}_{ij} $$

The indexes: i = person, j = day (− 1, 0, 1).

3. Pressure pain thresholds measured over 24 h at four time points: morning day 1 (provocation day), about 15 min after provocation, about 105 min after provocation, and in the morning the day after the provocation, day 2.
  Statistical model of pressure pain thresholds at Trapezius:
  $$ {Y}_{ijls}=\mu +{\alpha}_i+{\gamma}_s+{b}_1\ast time+{b}_2\ast localization+{b}_3\ast group+{b}_4\ast time\ast group+{b}_5\ast sex+{\varepsilon}_{ijls} $$

The indexes: i = person, j = time [1–4], l = localization [1–3].

The data were repeated measures, on individuals, over time and hence a dependency structure between observations were present. To handle this mixed effects linear models with a random intercept were used in all the above regression analyses [24]. Age and sex were considered potential confounders.

## Results

### Patient and control characteristics

The distributions of background variabels as gender, age, smoking, alcohol consumption and physical activity are, according to descriptive statistics different to some extent, between the pain group and the control group, Table 2. Pain related variables as number of pain sites, work percentage and sick leave, also shows distributional differences in descriptive statistics, which is expected, Table 2.

During the medical examination the patients reported following chronic diseases; asthma (*n* = 3), allergy (*n* = 5), hypertension (n = 3), hypercolesterolemi (*n* = 1), gastroesophageal reflux disease (n = 5), hypothyreosis (*n* = 2), migrain (*n* = 4), depression (n = 1), polycythemia vera (n = 1), and the controls reported asthma (n = 1), allergy (n = 1), and hypothyreosis (n = 1). Eleven of the patients and three of the controls stated that they had reached menopause. None of the female participants stated that they had ongoing menstruation during the medical examination.

The medications being taken by the patients with chronic pain included non-narcotic-containing analgesics (as required) (*n* = 18), narcotic-containing analgesics (as required) (*n* = 4), antidepressants (*n* = 9), sleeping pills (*n* = 2),bronchodilators (n = 2), inhaled corticosteroids (n = 2), thyroid hormones (n = 2), omeprazole (*n* = 5), beta blockers (n = 2), ACE inhibitors (n = 2) and hypolipidemic agents (n = 1). Medications being taken by the controls included non-narcotic-containing analgesics (as required) (*n* = 3), and thyroid hormones (n = 1), Hence, nine participants were on long term treatment with antidepressants; at the time of the examination there were no clinical signs of severe ongoing depression.

On the day of the provocation the participants were told to take medication as usual and the same day they were also asked about medication and coffee intake. Three patients had paracetamol the morning before provocation. Of the 26 persons in the pain group, 14 had coffee the morning before the provocation. In the control group 6 out of 12 had coffee in the morning.

### Pain intensity and perceived exertion during the provocation

**Fig. 1:**
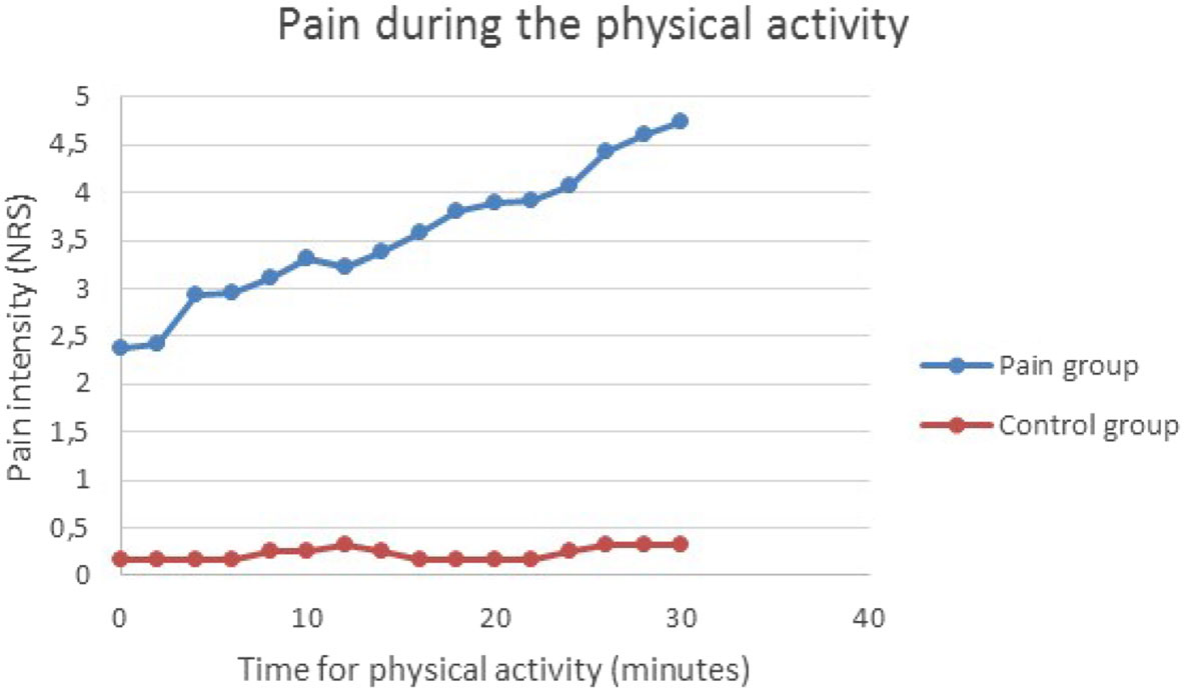
Pain intensity during the provocation**.** The arm-cycling began with a load of 100 g for women and 200 g for men and then increased by 200 g every 10 min up to 500 g for women and 600 g for men respectively. The figure shows model based means of pain intensity (NRS). The mean standard errors (SE) for the estimated model means (LSmeans) were 0.50 for the control group and 0.34 for the group with chronic neck pain. In the figure time is represented by a category variable to allow for any kind of pattern over time. The pattern is close to a linear relationship, why we in the final analysis let time be a continuous variable. In the final analysis the interaction time*group was also statistically significant (*p* < 0.001) and hence the development of pain intensity over time were different for the two groups

Pain intensity increased in the pain group during the arm cycling (Fig. 1). Details for the regression analysis are presented in Table 3.The regression model for pain intensity included the statistically significant variables *time*, as a continuous variable, and *group* (control or pain). The interaction time*group was also statistically significant and hence the development of pain intensity over time were different for the two groups. From the model, Table 3, we can get the estimated quantification of this. Hence, the pain intensity (NRS) in the pain group was estimated to increase from 2.65 to 4.68, while in the control group the pain intensity was estimated to be constant on the level 0.17. The median values in Rating of Perceived Exertion (RPE) were 10 in the control group and 14 in the pain group, respectively.

**Table 3:** Two regression analyses: 1) Pain intensity during the 30 min of provocation, assessment every second minute. 2) Evening pain intensity the day before the provocation, the evening of the provocation day and the evening 1 day after. Linear mixed effects models with a random intercept. Age and sex were checked for being possible confounders, but they did not qualify and were hence not adjusted for

Pain intensity (NRS)						
	During the provocation		Evening pain			
**N = 38**	**Parameter estimates** **(SE)**	**p-value**	***N*** **= 35**		**Parameter estimates** **(SE)**	**p-value**
**intercept**	2.58 (0.316)	<.0001	**intercept**		4.36 (0.427)	< 0.0001
**Time** (min)	0.07 (0.004)	<.0001	**Time**	1	−1.12 (0.417)	0.2356
				2	−0.08 (0.423)	
				3	0	
**Group:** control Reference: pain	−2.41 (0.555)	<.0001	**Group:** control Reference: pain		−3.88 (0.790)	< 0.0001
Time*Group: control Reference: pain	−0.07 (0.007)	<.0001	Time*Group: control Reference: pain		0.88 (0.800)	0.2738

### Evening pain intensity from diary

**Fig. 2:**
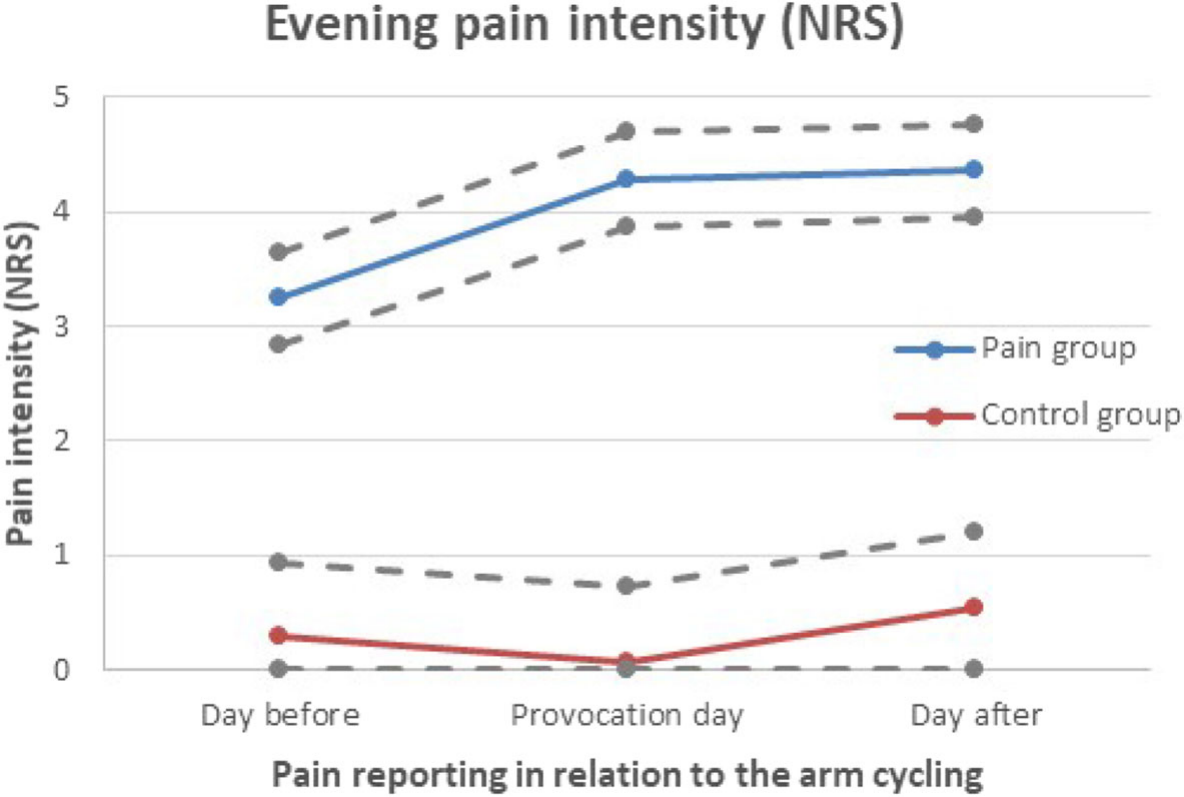
Evening pain intensity (NRS) reported in the diary; before, during and after the provocation. The figure shows model based means (EM means) of pain intensity (NRS), from the regression analysis. The dotted grey lines represents EM means ± standard error. The interaction time*group were not statistically significant (*p* = 0.2738) and hence the development of pain intensity over time were not proven to be different for the two groups

The chronic pain group showed increased pain intensity the two evenings following after the arm cycling, compared with baseline, (Fig. 2).

In the regression model for evening pain, the variable time were first used as a categorical variable. The evening pain modeled were from three time points; before the provocation (mean of seven previous evenings), evening of the provocation day, and evening the day after the provocation.

The regression model for pain intensity in evenings included the statistically significant variable group (control or pain). Time and the interaction time*group were not statistically significant, there was a tendency of increased pain intensity in the pain group in the evening after the arm cycling though not statistically significant, Fig. 2. Details for the regression analysis are presented in Table 3.

The Table 3 present the *p*-values connected to the parameters in the tested model. When the model includes a statistical interaction, as is the case here (time*group), the p-values for the main effects cannot be interpreted in themselfs. The Fig. 2 shows the development over time for the two groups, based on the model*.*

### Pressure pain thresholds before and after the provocation

**Fig. 3:**
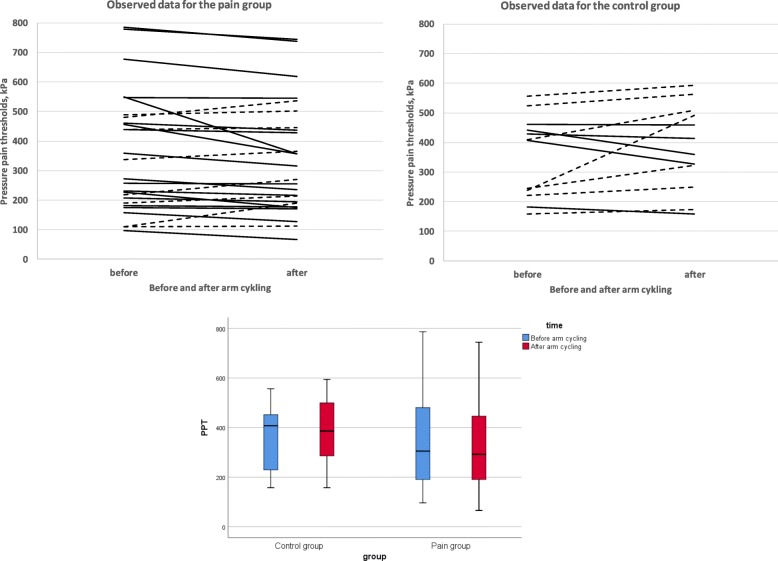
Note that this is a graph of raw data. Observed mean PPT at the right Trapezius before and 15 min after the provocation. Dotted lines represent individuals with increased PPT after exercise. Solid lines represent a decreased PPT, respectively. A box-plot is presenting the distribution of values before and after the arm cycling in the two group

The mean values, from the three locations, of the observed PPT at the right trapezius before and about 15 min after the armcycling are presented in Fig. 3.

Details for the regression analysis are presented in Tables 4 and 5. The regression model, analyzing all four time points, for PPT at trapezius included the statistically significant variables time (categorical) and location (in the model using the three measures at trapezius as separate measures). The interaction time*group were statistically significant and hence the development of PPT:s over time were different in the two groups. PPT:s had decreased in the pain group when measured about 15 min after the arm cycling, but had increased to its original level when measured about 105 min later, and was then basically maintained at the same level the day after, Tables 4 and 5. In the control group the PPT:s increased as expected immediately after, and then there were slightly different patterns at the three different measurement points, but overall there was a tendency for continued elevated thresholds after 105 min and the day after, Tables 4 and 5. The analysis using a mean of the three measurement points shows very similar results.

**Table 4:** Pressure pain thresholds at Trapezius, at four time points: before the provocation, 30 min after, 105 min after and 24 h after. P-values are presented for a mixed effects models including the interaction between time and group. The models include a random intercept, but side is also included as random effect. Age and sex were checked for being possible confounders, but only sex qualified and were adjusted for

Trapezius N = 38			
Three separate location		Mean of 3 locations	
	**p-value**		**p-value**
**intercept**	< 0.0001	**intercept**	< 0.0001
**Time**	< 0.0001	**Time**	< 0.0001
**Group** (pain, control)	0.7974	**Group** (pain, control)	0.7925
**Location**	< 0.0001		
Time*Group	< 0.0001	Time*Group	< 0.0001
Time*Group*Location	< 0.0001		
**Sex**	0.0043	**Sex**	0.0043

**Table 5:** Pressure pain thresholds at Trapezius. Here model based means (EM means) are presented as complement to the Table 4 with *p*-values. The EM means were checked for confounding by sex and age, but only sex needed to be adjusted for

Pressure pain thresholds at Trapezius, kPa ***N*** = 38					
		**Time in relation to arm cycling**			
	**Location**	**Before**	**15 min after**	**105 min after**	**Morning after**
**Pain group**	**EM means, Standard error 34.5**				
	**T1**	383	367	372	370
	**T2**	376	369	380	372
	**T3**	411	404	417	412
	**EM means, Standard error 43.5**				
**Control group**	T1	335	345	362	362
	T2	395	404	410	410
	T3	436	453	439	439

## Discussion

One main finding was that chronic neck-shoulder pain was associated with increased pain intensity during, and the following two evenings after the arm cycling despite the low perceived exertion. In contrast, absence of chronic pain, was associated with low or no pain during the arm cycling, and staying at this low level also the day after. These findings may be important to consider in pain rehabilitation and in assessment of physical work ability in chronic pain patients. In the scientific literature there appears to be sparse studies that illustrate pain intensity the day after a light physical exercise in this patient group, an exercise that could be similar to that which may occur in lighter manual work.

Secondly, patients with chronic neck-shoulder pain showed decreased pain thresholds (i.e. increased pain sensitivity) after a light physical exercise involving painful regions, indicating mechanical hyperalgesia and impaired EIH (Fig. 3, Tables 4 and 5). Previous studies have also shown impaired EIH in patients with chronic pain such as WAD [8], chronic musculoskeletal pain [9], knee osteoarthritis [25], chronic low back pain, and fibromyalgia [1–4]. However, a recent review/focus article concluded that the acute effects of exercise on EIH is variable in chronic pain populations and that not all chronic pain studies demonstrate an impaired EIH [11]. The mechanisms for EIH are poorly understood. As reviewed by Rice et al. several biological mechanisms have been explored e.g. the opioid system, the endocannabinoid system, the serotonergic system, the immune system and the autonomic nervous system. Also, psychological factors such as fear of pain, catastrophizing and beliefs of perceived threat may influence EIH. The increase pain intensity in the pain group could also be related to signs of temporal summation i.e., progressive increase in pain intensity as a function of repeated noxious stimulation [26, 27]. In clinical settings repetition-induced summation of activity-related pain (RISP) have been used to indicate temporal summation [26, 27]. As evident from Fig. 1 the pain intensity in the pain group increased at each load level during the provocation, which is consistent with other studies of patients with chronic pain [26, 27]. Lannersten and Kosek, have shown that segmental and plurisegmental pain inhibitory mechanisms were activated in patients with myalgia when they exercised parts of the body that did not have pain [2]. The controls showed increased thresholds indicating EIH, even after a light physical load (Fig. 3, Tables 4 and 5). In a meta-analytical review of the hypoalgesic effects of excercise, it appears that the EIH effects were generally transient and that the optimal dose of exercise required could not be determined with available data [10].

There are, as expected and also shown in Fig. 3, an individual variability in the PPT response after the physical load. Unfortunately the study sample is limited and therefore we are not able to identify any subgroups of EIH-respons. However, among the controls there were 7/12 (58%) having increased PPT after exercise (EIH), and among the chronic pain patients there were 18/26 (69%) having decreased PPT after exercise (hyperalgesia), indicating that there may be subgroups (Fig. 3). In future research, it might be interesting to study if there are subgroups of response patterns after exercise.

Thirdly, this study could not show that a light physical load had an impact on pain thresholds in the neck area the following day in patients with chronic neck-shoulder pain (Tables 4 and 5). There was a tendency in the control group, that pain thresholds, increased the following day (Tables 4 and 5). Is it possible for EIH to remain the day after a physical load? In addition, is it possible that chronic pain patients have a deterioration in the EIH, which increase the risk of pain the day after a light physical load?

To our knowledge, exercise induced hypoalgesia, with increased pain thresholds, have been studied primarily close to physical exercise, and in the scientific literature, there are to the best of our knowledge no studies reporting pain thresholds the following day.

### Limitations

When studying pain it is always important to have in mind the individual differences and the biopsychosocial interactions that form them [28]. There are relatively few participants in the study, which gives us a low precision in our estimates and limits the number of possible confounders we can adjust for. The distributions of background variabels as age, smoking, and education are to some extent different, between the pain group and the control group. We checked for age and sex confounding in the analyzes, but the sample size was to small to adjust for several possible counfounders. Some of the potential counfounders, as alcohol consumption, also had a large proportion of missing.

We have used mixed effects linear regression to analyze the change over time in outcomes. This regression method assumes that the utcome is continuous, and the residuals to have a normal distribution. Pain intensity measured with the scale NRS is an ordinal variable, and hence not continous. Pain thresholds are continuous, but truncated. We are aware of the pragmatic approch we have applied and urge the reader to consider analysis to mainly detect associations and rough patterns, but not to in detail interpret the size of changes or differences.

An issue overseen in most analysis of pressure pain thresholds, is that calculating the difference between two values, were at least one is truncated, is problematic. Analyzing the data with a mixed effects model handles that problem, but still has the problem of possibly not normally distributed residuals. Both these issue, ordinal and truncated outcomes, could possibly be handled by using logistic quantile regression respectively linear quantile regression. Though, quantile regression requires large sample sizes, which is not the case here.

Another important methodological issue seldom discussed is how to handle the three trapezius measures. One could see them as three separate locations and analyzed them as separate measurements, as in the main analyses in this paper. Or one could, as is common, take the mean of the three locations and use that as a trapezius measure, hence with better precision. We performed both analysis in this paper and find no major differences in results, but we argue that this should be further investigated in larger studies.

## Conclusions

Having chronic neck-shoulder pain was associated with increased pain intensity during and the day after a light physical exercise involving painful regions, an exercise which could be similar to that wich may occur in lighter manual work. In addition, they showed decreased pain thresholds close to the exercise, indicating mechanical hyperalgesia and impaired excercise induced hypoalgesia. These findings could be important to take into account in work ability assessments. However, this study could not show that a light physical load, had an impact on pain thresholds in the neck the following day, in patients with chronic neck-shoulder pain. Nevertheless, there was a tendency for increased pain thresholds in the control group, which may reflect exercise induced hypoalgesia the day after exercise.

## Data Availability

The study materials has ethical and legal restrictions on sharing a de-identified data set. All data including questions about the health of individuals are obligated to follow “The General Data Protection Regulation” in EU. We are not allowed to freely share the data without additional permission from the ethics committee for the specific purpose (Regionala Etikprövningsnämnden i Göteborg).

## Abbreviations

Borg RPE scale: Borg rating of perceived exertion scale
HADS: Hospital anxiety and depression scale
IQR: Interquartile range
NRS: Numeric rating scale
PPT: Pressure pain threshold
SAS: Statistical analysis software
SD: Standard deviation
SMBQ: Shirom–Melamed burnout questionnaire
WAD: Whiplash associated disorders
